# Afro-Latin American Pharmacogenetics of *CYP2D6*, *CYP2C9*, and *CYP2C19* in Dominicans: A Study from the RIBEF-CEIBA Consortium

**DOI:** 10.3390/pharmaceutics16111399

**Published:** 2024-10-30

**Authors:** Mariela Guevara, Fernanda Rodrigues-Soares, Carla González de la Cruz, Fernando de Andrés, Ernesto Rodríguez, Eva Peñas-Lledó, Adrián LLerena, CEIBA Consortium of the Ibero-American Network of Pharmacogenetics and Pharmacogenomics RIBEF

**Affiliations:** 1 Facultad de Ciencias de la Salud, Universidad Nacional Pedro Henríquez Ureña, Santo Domingo 10514, Dominican Republic; ernest2288@yahoo.es (M.G.); er18-2087@unphu.edu.do (E.R.); 2 Faculty of Medicine and Health Sciences, University Institute for Bio-Sanitary Research of Extremadura INUBE, University of Extremadura, 06006 Badajoz, Spain; fernanda.soares@uftm.edu.br (F.R.-S.); carla.gonzalezd@externos.salud-juntaex.es (C.G.d.l.C.); elledo@unex.es (E.P.-L.); 3 Department of Pathology, Genetic and Evolution, Universidade Federal do Triângulo Mineiro, Uberaba 38025-350, Brazil; 4 Department of Analytical Chemistry and Food Technology, Faculty of Pharmacy, University of Castilla-La Mancha, 02008 Albacete, Spain; fernando.deandres@uclm.es

**Keywords:** *CYP2D6*, *CYP2C9*, *CYP2C19*, Dominican, African, ancestry

## Abstract

**Background/Objectives**: Research on pharmacogenetic variability in response to prescribed drugs and across ethnic groups is essential for personalized medicine, particularly in admixed and unstudied populations. For the first time, this study examines *CYP2D6*, *CYP2C9*, and *CYP2C19* alleles and genotypes in 197 healthy volunteers from the Dominican Republic, as part of the RIBEF-CEIBA collaborative network. **Methods**: The analysis focuses on the participants’ tri-hybrid genomic ancestry, with CYP alleles determined by real-time PCR and molecular ancestry inferred using 90 AIMs. Linear regression was used to associate ancestry components with CYP frequencies. **Results**: The average ancestry was 23.8% European, 42.6% Native American, and 33.6% African, the latter being higher than in most Latin American populations. Native American ancestry was also higher than expected. Predicted phenotype frequencies based on genotypes were 4.2% poor metabolizers (gPMs) and 3.6% ultrarapid metabolizers (gUMs) for CYP2D6, as well as 3% gPMs, 22.8% rapid metabolizers (gRMs), and 1.5% gUMs for CYP2C19. No gPM individuals were observed for CYP2C9. Certain alleles associated with decreased CYP2D6 activity (*17 and *29) and increased CYP2C19 activity (*17 and gUMs) were positively linked with African ancestry and negatively with Native American ancestry. Rare *CYP2C9* alleles (*5 and *6) with clinical relevance were additionally found. **Conclusions**: These findings build on previous results from the RIBEF-CEIBA collaborative network, demonstrating differences in allele frequencies of *CYP2D6*, *CYP2C9*, and *CYP2C19* in relation to genomic ancestry. In summary, ethnicity must be considered in the development of pharmacogenetic guidelines for clinical application, research, and regulation to avoid widening the biotechnology gap and to allow Personalized Medicine to reach the entire world population.

## 1. Introduction

The Ibero-American population presents a great diversity of pharmacogenetic (PGx) polymorphisms due to interbreeding, population structure, and migration [1]. However, the study of PGx biomarkers in such mixed countries still needs to be explored, particularly in specific, unstudied, or underrepresented populations like the Dominican Republic. The present study aims to replicate the previous results of the RIBEF-CEIBA (Red Iberoamericana de Farmacogenética y Farmacogenómica) Consortium by expanding with a population with a presumably larger Afro-Latin American component that is generally underrepresented in pharmacogenetic studies [2,3].

In this context, it is important to highlight that Caribbean populations present a different structure from mainland populations because their African ancestry is higher [4]. This is likely due to the region’s proximity to the North Atlantic Ocean, which facilitated nautical contact with the African coast [5]. Furthermore, Hispaniola island, where the Dominican Republic and Haiti are located, holds historical significance as the first place Europeans arrived, making admixture events earlier than in other parts of the Americas. Historically, by the XVI century, Native American inhabitants from Hispaniola, the Taínos, were decimated by Europeans, and the influx of African slaves compensated for this reduction in the Native population. It is also reported that Native American survivors gathered with escaped African slaves to form communities [6].

Against this backdrop, the efforts of the RIBEF-CEIBA Consortium are particularly relevant. This consortium was created to characterize interindividual and interethnic variability of Ibero-American populations regarding the polymorphic *CYP2D6*, *CYP2C9*, and *CYP2C19* drug-metabolizing enzyme genotypes and phenotypes. This is crucial, especially because these enzymes are involved in the metabolism of many commonly prescribed drugs [7]. Specifically, CYP2D6 is responsible for metabolizing up to 25% of commonly used drugs in clinical practice, such as codeine and other opioids, antidepressants, antipsychotics, and tamoxifen. Meanwhile, CYP2C9 is involved in the metabolism of warfarin, phenytoin, and NSAIDs, whereas CYP2C19 is crucial for clopidogrel, antidepressants, and proton pump inhibitors metabolism [2,3].

Indeed, genetic polymorphisms of the genes encoding for these enzymes were studied by this Consortium in 6060 healthy volunteers from different countries across North, Central, and South America. The studied populations were classified according to their self-reported ancestry: 1395 Native Americans, 2571 Admixed Latin Americans, 96 Afro-Latin Americans, 287 white Latin Americans (from Cuba), 1537 Iberians, and 174 Ashkenazi Jews. The results indicated that Native Americans had higher frequencies of wild-type alleles for all genes but lower frequencies of CYP2D6*41, CYP2C9*2, and CYP2C19*17, as well as fewer *CYP2C19* genetic ultrarapid metabolizers (gUMs) than the rest of the sample population studied [2]. Therefore, understanding this ancestry-related information is essential for developing and updating appropriate pharmacotherapy recommendations from a public health perspective [2,3,8,9,10].

Turning specifically to the Dominican Republic, it is notable that approximately 23.5% of individuals self-identify as “mestizos”, 23.1% as “mulatto”, 20.4% as Black, 14.9% as White, and 10.8% as “Indigenous” [11]. However, despite studies showing even with molecular ancestry evidence that the Dominican and other Caribbean populations present predominantly African and European ancestry [5,12,13,14], cultural factors often lead Dominicans to fail to recognize and identify with their African ancestry [15]. Consequently, more studies like the present one on the genomic ancestry of the Dominican population, which also combines, for the first time, genomic ancestry with a pharmacogenetic approach, are needed.

Interestingly, the genomic ancestral component of the previously studied RIBEF-CEIBA population [2] was investigated in 3387 healthy volunteers using 87 ancestry informative markers (AIMs) [3]. The molecular ancestry results aligned with self-reported ancestry, showing that the CYP2C19*17 allele and CYP2C19 gUMs (increased-activity allele and predicted ultrarapid phenotype) were negatively associated with Native American ancestry. Conversely, CYP2D6*41 and CYP2C9*2 (decreased-activity alleles) were positively associated with European ancestry, while CYP2D6*17 and CYP2D6*29 (decreased-activity alleles) were positively linked to African ancestry.

Given the limited representation of Afro-Latin American ancestry in previous pharmacogenetic studies in general and of RIBEF-CEIBA in particular, it is necessary to target populations with a potentially high percentage of African ancestry, such as the Dominican Republic.

To address this gap, the present study analyzes, for the first time, the main genetic polymorphisms of *CYP2D6*, *CYP2C9*, and *CYP2C19* in the Dominican Republic population and their relation to genomic ancestry, in the context of the RIBEF-CEIBA study protocols.

## 2. Materials and Methods

The study included 197 healthy unrelated Dominican students and staff recruited from the “Universidad Nacional Pedro Henríquez Ureña” (UNPHU, https://unphu.edu.do, accessed on 27 September 2024) in Santo Domingo, Dominican Republic. None of the participants were immigrants, which applied to at least two previous generations. The study adhered to the principles outlined in the Declaration of Helsinki for human research and was approved by the *Consejo Nacional de Bioética en Salud* Ethical Committee (018-2022). Written informed consent was obtained from all participants prior to sample collection.

### 2.1. CYP2D6 Genotyping and Predicted Phenotype Inferences

Analysis of CYP2D6*2, *3, *4, *6, *9, *10, *17, *29, *35, and *41 was performed using commercially available genomic DNA Taqman^®^ assays (Applied Biosystems, Foster City, CA, USA). *CYP2D6* genotypes were assigned according to the presence of “key” SNPs associated with the alleles of interest (Table 1). All assays included negative (no DNA) and positive (heterozygous and/or homozygous) control samples from previous studies of our group. Plates were read with an ABI 7300 instrument and QuantStudio 5 (Applied Biosystems, Foster City, CA, USA), and the following thermocycling conditions were applied: 10 min for initial denaturation at 95 °C, followed by 40 denaturation cycles of 15 s at 92 °C and annealing at 60 °C for 1 min. Allele discrimination was performed for 30 s at 60 °C.

XL-PCR was performed to determine whether individuals carry the *CYP2D6* gene duplications or CYP2D6*5 gene deletion, as described previously [16]. To predict the enzyme activity, an activity score (AS) was assigned to each *CYP2D6* allele: *CYP2D6-wt*, *2, or *35 were assigned as normal activity alleles (AS = 1); CYP2D6*3, *4, *5, and *6 as no function alleles (AS = 0), *17 and *29 variants were assigned as 0.5 due to their association to decreased activity; CYP2D6*9, *10, and *41 alleles were assigned as 0.25 as they are related to almost absent enzymatic activity; and the multiplications of the active alleles CYP2D6*1xN or *2xN were assigned as AS = 2. Each individual AS value is calculated as the sum of each allele AS. Thus, according to the most recent classification [17], individuals with zero *CYP2D6* active genes (AS = 0) were classified into poor metabolizers (gPMs), and those with more than two active genes (AS > 2.25) were categorized into ultrarapid metabolizers (gUMs), while individuals with the AS from 0.25 to 1 were classified as intermediate metabolizers (gIMs) and the remaining individuals (AS from 1.25 to 2.25) were classified as normal metabolizers (gNMs).

### 2.2. CYP2C9 Genotypes and Predicted Phenotype Inferences

CYP2C9*2, *5, *8 (decreased function), *3, and *6 (no function) analysis was performed externally at the Research Support Services of the University of Extremadura (SAIUEx) using commercially available Taqman^®^ assays. *CYP2C9* genotypes were assigned according to the presence of SNPs associated with the alleles of interest (Table 1). *CYP2C9* phenotypes can be predicted based on the genotype as follows: gPMs (two no-function alleles or one no-function plus one decreased function allele), gIMs (two decreased function alleles or one normal function plus one decreased- or no-function allele), and gNMs (two normal-function alleles) [18].

### 2.3. CYP2C19 Genotypes and Predicted Phenotype Inferences

CYP2C19*2, *3, *4, *5 (no function), and *17 (increased function) genotyping was performed externally at the Research Support Services of the University of Extremadura (SAIUEx) using commercially available Taqman^®^ assays. *CYP2C19* genotypes were assigned according to the presence of SNPs associated with the alleles of interest (Table 1). Individuals were classified into gPMs (two no-function alleles), gIMs (one no-function allele combined with a non-no function allele), gNMs (two normal function alleles), rapid metabolizers (gRMs) (one normal function allele plus one increased function allele), and gUMs (two increased function alleles) [19].

#### 2.3.1. Continental Ancestry Analysis

African, European, and Native American individual ancestry were estimated in 178 Dominican individuals by genotyping 90 ancestry informative markers (AIMs) from the same panel as standardized in the previous RIBEF-CEIBA study, as it is known that panels with more than 80 AIMs provide accurate estimates of continental admixture in Latin Americans [3,20]. AIMs genotyping was performed at the Spain National Genotyping Center (CEGEN) from Santiago de Compostela, using iPLEX assays followed by mass spectrometry analysis using the MassARRAY System (Agena Bioscience, San Diego, CA, USA). The admixture values were then inferred using the model-based method implemented in Admixture software v. 1.3.0 [21], assuming a tri-hybrid model (K = 3) and performing an unsupervised analysis, using 114 Spaniards and 296 Peruvian Native Americans from the RIBEF-CEIBA database as parental European and Native American parental populations [3], as well as 209 African Yoruba individuals from 1000 Genomes Project as the African parental population [22].

The ancestry analysis for the previous RIBEF-CEIBA population was described previously [3], and these results were merged with data from the Dominican Republic to create an updated database and perform the linear regression analysis.

#### 2.3.2. Data Analyses

Allele frequencies of *CYP2D6*, *CYP2C9*, and *CYP2C19* were calculated using the Adegenet package v. 2.1.10 [23], and ancestry individual proportions were plotted with the *barplot* function in R Platform [24]. A chi-squared test was performed in the R platform to compare allele frequencies among populations comprising their country or self-reported ancestry, and *p*-values lower than 0.05 were considered significant. To describe the dependence of CYP allele frequencies on the three ancestry components, the linear coefficient beta, its significance (*p*-value), and the percentage of CYP allele frequencies variance, explained by each continental ancestry (R^2^), were estimated by a linear regression analysis using the *lm()* function in R platform. For linear regression, *p*-values lower than 0.01 were regarded as statistically significant.

## 3. Results

**Figure 1:**
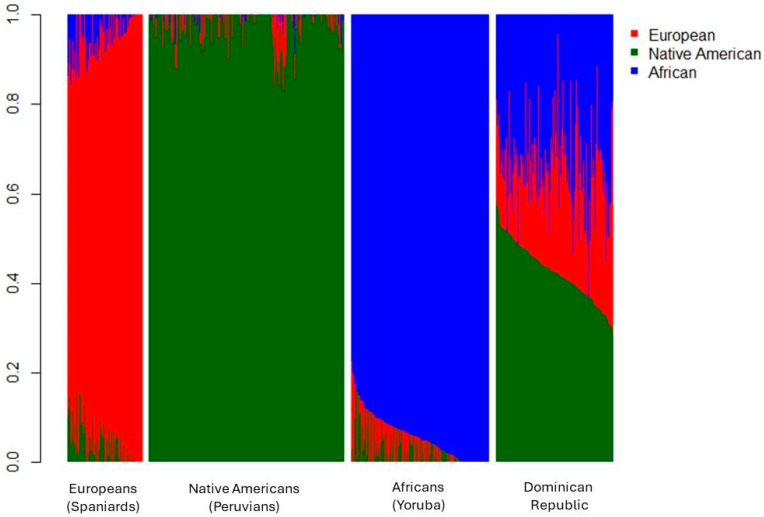
Ancestry of individuals from the Dominican Republic, along with reference parental populations included in the analysis. Europeans: Spaniards from Extremadura, Spain; Native Americans: Indigenous populations from Peru (Ashaninka, Aymara, and Shimaa ethnic groups); Africans: Yoruba from Nigeria, as obtained from the 1000 Genomes Project.

Of the 197 individuals, 64.5% were women and 67.5% were students from UNPHU. Figure 1 shows the individual tri-hybrid ancestry proportions for Dominican Republic individuals (n = 178) and the parental populations used in the analysis.

Of the Dominican individuals genotyped for genomic ancestry, 155 were self-identified as admixed (34% average genomic African ancestry), 12 as White (28% average genomic African ancestry), and 11 as Afro-descendant (39% average genomic African ancestry).

The present results indicate that the average genomic ancestry for the 178 Dominican individuals was 23.8% European, 42.6% Native American, and 33.6% African. This demonstrates that the population is highly admixed, with over 75% of their non-European ancestry (AFR and NAT). Notably, this population ranks fourth in African ancestry among the 33 Latin American populations studied by RIBEF [3], with nine individuals exceeding 50% African ancestry (maximum 63%). Interestingly, none of these individuals self-identified as Afro-descendant.

Table 2 shows the allele and predicted phenotype frequencies of *CYP2D6*, *CYP2C9*, and *CYP2C19* in the Dominican Republic. The CYP2D6*39 allele was detected in one individual, assigned by the presence of only one variant (rs1135840) of the CYP2D6*2 allele. The regression analysis between the three ancestry components and CYP allele frequencies performed in the previous study from our group was updated, including the Dominican Republic data (Supplementary Table S1). Regarding *CYP2D6*, the *6 allele was added, although none of the associations were significant for it. Moreover, the significant association between the frequency of CYP2D6 gPMs and European and Native American ancestry and the association of the *10 allele with EUR ancestry was no longer observed. The remaining results have not changed, although statistical power, in general, has increased, so the significant associations were *4 positively associated with European (R^2^ = 0.22; *p* < 0.01) and *10, *17 and *29 alleles positively associated with African ancestry (R^2^ = 0.32, 0.94 and 0.88; *p* < 0.001) and negatively related to Native American ancestry (R^2^ = 0.31, 0.24 and 0.27; *p* < 0.01) (Supplementary Table S1).

With regard to *CYP2C9* and *CYP2C19*, the new regression analysis included CYP2C9*6, CYP2C19*3, *4, and *5; however, none showed significant associations (Supplementary Table S2). The remaining results are consistent with the previous analysis, showing significant associations. CYP2C9*2, *3, and gPMs were positively associated with European ancestry (R^2^ = 0.84, 0.22, and 0.29; *p* < 0.01) and negatively associated with Native American ancestry (R^2^ = 0.7, 0.2, and 0.25, *p* < 0.01). Additionally, CYP2C19*17 and gUMs were positively associated with both European (R^2^ = 0.67 and 0.68; *p* < 0.0001) and African ancestry (R^2^ = 0.34 and 0.32; *p* < 0.001), while showing a negative association with Native American ancestry (R^2^ = 0.94 and 0.92; *p* < 0.0001) (Supplementary Table S2).

## 4. Discussion

Here, the first pharmacogenetics study including genomic ancestry and *CYP2D6*, *CYP2C9*, and *CYP2C19* allele and genotype frequencies for the Dominican Republic population is reported. Pharmacogenomic recommendations are frequently updated by regulatory agencies (i.e., the U.S. Food and Drug Administration (FDA), the European Medicines Agency (EMA), and the Agencia Española de Medicamentos y Productos Sanitarios (AEMPS), which provide drug labeling information for numerous active principles and biomarkers [25,26,27]. Nevertheless, pharmacogenomic drug labeling and guidelines from consortia like the Clinical Pharmacogenetics Implementation Consortium (CPIC) and the Dutch Pharmacogenomics Working Group (DPWG) may not completely meet the needs of certain patient groups such as those from Latin America. This highlights the necessity of further stratifying individuals at risk and considering alternative strategies before the clinical implementation of personalized drug treatments. As a result, the concepts of population pharmacogenomics, genetic ancestry, and ethnicity have become crucial. The analysis of these understudied populations could reveal novel genetic variants, which would aid in the more precise stratification of these groups.

The population of the Dominican Republic is remarkably heterogeneous, with Native American, European, and African components, the last being higher than most Latin American populations. Regarding self-reported ancestry, most of the individuals (87%) were self-reported Admixed, which is more than the previously described for the full population of this region (46.6% if considering mestizos and mulattos) [11]. Considering molecular ancestry results, in our sample, Native American and African components were predominant, contrasting previous studies that reported a predominance of European and African ancestry components in this population [4,5]. A previous genomic study performed individual and local ancestry in Caribbean populations, including the Dominican Republic, and found limited pulse events from the European population, which may have resulted in a founder effect. In contrast, African migration has occurred more constantly, with two main migration pulses: the first around 15 generations ago, corresponding to 3–16% of the Atlantic slave trade, and the second around seven generations ago, corresponding to more than half of the slave trade. Additionally, a single pulse of Native American ancestry is detected, and their tracts are shorter than any other ancestry (thus, older), showing a rapid decimation of this population on this island. On the other hand, mainland populations have received repeated European migration events, giving both regions different genetic structures [5].

The contrasting results could be attributed to differences in sample composition. The current study included 178 individuals from Santo Domingo, whereas the previous research examined 34 Dominican individuals from South Florida and 27 from New York (total N = 61). In addition to the variations in sample size and geographic location, the previous study conducted ancestry analyses using approximately 390K SNPs, while the present study employed 90 AIMs to assess tri-hybrid individual ancestry.

In general, Dominicans present a frequency similar to other Afro-Latin American populations (*p* > 0.05), such as those from Costa Rica and Cuba, especially when considering the CYP2D6*17 and CYP2D6*29, previously associated with African ancestry [3]. The only exception was CYP2D6*2, significantly lower in populations from Costa Rica than in Dominican individuals (*p* = 0.03) [3]. About *CYP2C9* and *CYP2C19*, Dominican subjects present a frequency similar to other Afro-Latin American populations (*p* > 0.05), such as those from Costa Rica and Cuba, except for CYP2C9*2, significantly lower in Costa Rican than in Dominicans (*p* = 0.03).

The CPIC has published 28 therapeutic recommendation guidelines that are used as reference for pharmacogenetics implementation worldwide [28]. However, these guidelines are developed based on evidence mostly from European populations. In total, 14 out of the 28 published guidelines present recommendations for *CYP2D6*, *CYP2C9*, and/or *CYP2C19* genotypes, but only one presents ethnically specific recommendations, the warfarin guideline [29]. This guideline provides specific recommendations for African and Afro-American populations due to the higher frequency of certain *CYP2C9* alleles (*5, *6, *8, and *11) that reduce enzyme activity. Warfarin dosing algorithms offer better predictions compared to the FDA table alone; however, for Afro-descendant populations, algorithms that only consider CYP2C9*2 and *3 alleles are not more effective than those that do not incorporate genetic data. The guideline addresses clear recommendations, stating that if *5, *6, *8, and *11 alleles have not been genotyped, warfarin should be dosed clinically without considering genetic information. It also advises against pharmacogenetic testing in such cases, as dosing based solely on CYP2C9*2 and *3 alleles may lead to inadequate and falsely reassuring International Normalized Ratio (INR) monitoring. This presents a challenge in implementation, as most clinical laboratories conduct FDA-approved tests that include only the CYP2C9*2 and *3 alleles. However, some laboratories have begun offering validated expanded panels [29].

In this study, genotyping of CYP2C9*5, *6, and *8 was performed in Dominican individuals. Table 3 compares the frequencies among RIBEF-CEIBA Dominicans, self-reported Black Cubans, and other African/Afro-Americans described in PharmGKB [30]. It is interesting to notice that, in general, the frequencies of these alleles are similar among sub-Saharan Africans and Afro-Americans [30]. Ancestry mirrors the demographic history of the Caribbean islands, which exhibit a higher proportion of sub-Saharan African ancestry.

In the Cuban population, CYP2C9*6 was absent. Likewise, CYP2C9*8 was not found in the Dominican samples included, while CYP2C9*5 was more than 10 times more frequent in Dominicans and Cubans when compared to the European population but lower than other Afro-American populations. Moreover, Dominicans present a CYP2C9*6 frequency more than 10 times higher when compared to Europeans and 5 times higher than in other Latino populations, but lower than other Afro-American populations, and the same is true for CYP2C9*8 in Cubans. These intermediate frequencies are concordant with the admixture of this population because Dominican individuals present a Native American component that other Afro-Americans do not, who usually bear only African and European components.

This heterogeneity is caused by admixture and besides star alleles’ intermediate frequencies, there may be other unknown genetic variants. Latin American populations are neglected in genomic studies, resulting in a huge gap in what is known about these populations. Pharmacogenetic tests are still developed for European populations because the evidence used to design these tests comes from these populations. However, genetic variation is very wide, and while Latin American and other neglected populations were not sequenced or studied in a proper depth, precision medicine will always be out of reach for these individuals, even if they have the financial resources to perform it. In this case, genome sequencing of Latin American and African populations may help to fill the gap of finding variants with possible clinical implications in pharmacogenetics and translational science.

As previously mentioned, different implementation guidelines include genotyping with *CYP2D6*, *CYP2C19*, and *CYP2C9*; only the previously mentioned *CYP2C9* and warfarin genotyping include ethnicity. Regarding *CYP2D6* and *CYP2C19*, many clinical guidelines highlight the significance of genotyping before prescribing certain drugs, such as tricyclic antidepressants like amitriptyline. One guideline suggests choosing a different drug for people with an intermediate *CYP2D6* phenotype and a rapid or ultrarapid *CYP2C19* phenotype [32]. Additionally, antidepressants such as citalopram/escitalopram from the serotonin reuptake inhibitor group should not be prescribed to individuals with CYP2C19*1/*17 or CYP2C9*17/*17 genotypes [33]. In line with the clinical repercussions that the implementation of the *CYP2D6* and *CYP2C19* genetic polymorphisms may have, it is worth noting that they are involved not only in the antidepressants mentioned but also in others such as venlafaxine [34] or antipsychotics such as risperidone [35], frequently used in combination with antidepressants. In summary, the optimization of *CYP2D6* and *CYP2C19* genotyping is relevant for the implementation in cases as severe and clinically important as suicide [36,37] or psychosis [38]. Optimization in polytherapy by considering CYP2D6 and CYP2C19 interaction, as well as other genes that act simultaneously in the metabolism of a drug, is extremely important for programs under development for clinical implementation [39].

This study presents some limitations, such as the low number of individuals in the Dominican population and, even when gathering the results of all RIBEF-CEIBA Consortium, data were not sufficient to associate the ancestry components with the gPM phenotype of none of the three CYPs. Moreover, the genetic screening in this study was restricted to a limited number of variants, making it valuable to a broader genomic approach in the future. However, as the first pharmacogenetic study of healthy volunteers in the Dominican Republic, which also includes the genomic ancestry of these individuals, these findings provide relevant pharmacogenetic biomarker information for the future implementation of pharmacogenetics and personalized medicine in this population to adjust doses and avoid adverse reactions correctly.

The variability in molecular ancestry may partly explain the results previously found in RIBEF network studies, which demonstrated a lack of prediction of the CYP2D6, CYP2C19, and CYP2C9 metabolic phenotype using the panels and genotyping schemes set out in the impregnation guidelines. If the genotype does not predict the phenotype in Mexico, Ecuador, or Nicaragua [10,40,41], which would be along the lines postulated above regarding the existence of ‘phenocopies’, since the phenotype is a consequence of a gene–environment interaction [42]; thus, other variables related to American ancestry should be considered.

Therefore, in light of the present results and previous studies, ethnicity should be considered when developing pharmacogenetic guidelines for clinical implementation, research, and regulation. This consideration is essential to prevent widening the biotechnology gap and to ensure that Personalized Medicine reaches the entire global population [43,44]. The inclusion of ethnicity in clinical research and pharmacogenetics was the basis for the Declaration of Mérida/T’Hó made by RIBEF in collaboration with CIOMS [45].

## Figures and Tables

**Table 1:** *CYP2D6*, *CYP2C9*, and *CYP2C19* variants and their corresponding Taqman^®^ assays utilized for Real-Time PCR genotyping in the Dominican population.

CYP Gene	CYP Alleles	rs ID	Nucleotide Change	Allele Functional Status	Taqman^®^ Assay ID
*CYP2D6*	*2	rs16947	2851C>T	Normal	C__27102425_10
		rs1135840	4181G>C		C__27102414_10
	*3	rs35742686	2550delA	None	C__32407232_50
	*4	rs3892097	1847G>A	None	C__27102431_D0
	*6	rs5030655	1707T>del	None	C__32407243_20
	*9	rs5030656	2616delAAG	Decreased	C__32407229_60
	*10	rs1065852	100C>T	Decreased	C__11484460_40
	*17	rs28371706	1022C>T	Decreased	C___2222771_A0
	*29	rs59421388	3184G>A	Decreased	C__34816113_20
	*35	rs769258	31G>A	Normal	C_27102444_F0
	*41	rs28371725	2989G>A	Decreased	C__34816116_20
*CYP2C9*	*2	rs1799853	3608C>T	Decreased	C_25625805_10
	*3	rs1057910	42614A>C	None	C_27104892_10
	*5	rs28371686	42619C>G	Decreased	C_27859817_40
	*6	rs933213	10601delA	None	C__32287221_20
	*8	rs7900194	3627G>A	Decreased	C__25625804_10
*CYP2C19*	*2	rs4244285	19154G>A	None	C__25986767_70
	*3	rs4986893	17948G>A	None	C__27861809_10
	*4	rs28399504	1A>G	None	C__30634136_10
	*5	rs56337013	90033C>T	None	C__27861810_10
	*17	rs12248560	−806C>T	Increased	C____469857_10

**Table 2:** Allele and predicted phenotype frequencies of *CYP2D6*, *CYP2C9*, and *CYP2C19* in the population studied from the Dominican Republic.

*CYP2D6* (n = 190)						*CYP2C9* (n = 195)		*CYP2C19* (n = 197)	
Allele	Frequency (NC)	Allele	Frequency (NC)	Predicted Phenotype	Frequency (NI)	Allele	Frequency (NC)	Allele	Frequency (NC)
*wt*	0.342 (130)	*29	0.047 (18)	gUM	0.036 (7)	*wt*	0.842 (328)	*wt*	0.662 (261)
*2	0.213 (81)	*35	0.016 (6)	gNM	0.637 (121)	*2	0.120 (47)	*2	0.173 (68)
*3	0.011 (4)	*39	0.003 (1)	gIM	0.284 (54)	*3	0.031 (12)	*17	0.165 (65)
*4	0.087 (33)	*41	0.061 (23)	gPM	0.042 (8)	*6	0.005 (2)	**Predicted phenotype**	**Frequency (NI)**
*5	0.039 (15)	*wtx2*	0.005 (2)			*5	0.003 (1)	gUM	0.015 (3)
*6	0.005 (2)	*wtx3*	0.005 (2)			**Predicted phenotype**	**Frequency (NI)**	gRM	0.228 (45)
*9	0.024 (9)	*2x2	0.013 (5)			gNM	0.719 (141)	gNM	0.442 (87)
*10	0.018 (7)	*4x2	0.021 (8)			gIM	0.276 (54)	gIM	0.284 (56)
*17	0.089 (34)					gPM	0 (0)	gPM	0.03 (6)

NC = Number of chromosomes. NI = Number of individuals.

**Table 3:** CYP2C9*5, *6, and *8 allele frequencies among the Dominican Republic and Cuban populations from RIBEF (bold) and African American PharmGKB-described populations [30].

*CYP2C9* Allele	AFR CPIC	AFR UKBB	AFR AOU	DR	CUB	LAT	EUR	EAS
*5	1.16%	0.91%	1.06%	**0.3%**	**0.4%**	0.87%	0.02%	0.00%
*6	0.85%	1.17%	1.10%	**0.5%**	**0.0%**	0.1%	0.03%	0.00%
*8	5.9%	5.11%	5.68%	**0.0%**	**2%**	0.74%	0.18%	0.37%

AFR CPIC: Afro-American as described by CPIC; AFR UKBB: Self-reported African American/Afro-Caribbean by UK Biobank; AFR AOU: Subsaharian African from All of Us Project; DR: Dominican Republic (present study); CUB: Cubans from RIBEF [3,31]; LAT: Latino described by CPIC; EUR: European described by CPIC; EAS: East Asian described by CPIC.

## Data Availability

The raw data supporting the conclusions of this article will be made available by the authors upon request.

## Supplementary Materials

The following supporting information can be downloaded at https://www.mdpi.com/article/10.3390/pharmaceutics16111399/s1, Table S1: Linear regression parameters of the *CYP2D6* alleles and predicted phenotype frequencies and tri-hybrid genomic ancestry; Table S2: Linear regression parameters of the *CYP2C9* and *CYP2C19* alleles and predicted phenotype frequencies and tri-hybrid genomic ancestry.

## Appendix A. CEIBA Consortium Affiliations

Graciela E. Moya ^1,^*, Eduardo Tarazona-Santos ^2,3,^*, Piedad Sarmiento ^4,^*, Angélica Borbón ^5,^*, Carolina Céspedes-Garro ^6,^*, Luis R. Calzadilla ^7,^*, Idania Rodeiro ^8,^*, Diadelis Remirez ^9,^*, Enrique Terán ^10,^*, Rocío Ortiz-López ^11,^*, Augusto Rojas-Martinez ^11^, Marisol López-López ^12,^*, Alberto Ortega-Vázquez ^12^, Martha Sosa-Macías ^13,^*, Carlos Galaviz-Hernández ^13^, Ronald Ramírez-Roa ^14,^*, Catalina Altamirano Tinoco ^15^, Manuela Grazina ^16,^*, Pedro Dorado ^17^, Humberto Fariñas ^18^, Francisco E. Estévez-Carrizo ^19,^*

* Group leader.

1 UCA Universidad Católica Argentina, Buenos Aires, Argentina.
2 UFMG Universidade Federal de Minas Gerais, Belo Horizonte, Brazil.
3 UPCH Universidad Peruana Cayetano Heredia, Lima, Perú.
4 Former: PUJ Pontificia Universidad Javeriana, Bogotá, Colombia; Present: Advanced Innovative Partners-AIP, Surfside, FL, USA.
5 Former: PUJ Pontificia Universidad Javeriana, Bogotá, Colombia; Present: Subdirección de Innovación en Salud Publica, Instituto Nacional de Salud de Colombia, Bogotá, Colombia.
6 Former: UCR Universidad de Costa Rica; Present: CEC ACIB-FUNIN, San José, Costa Rica.
7 Centro Comunitario de Salud Mental Habana Vieja, La Habana, Cuba.
8 ICIMAR Instituto de Ciencias del Mar, La Habana, Cuba.
9 CECMED Centro para el Control Estatal de la Calidad de los Medicamentos, Equipos y Dispositivos Médicos, La Habana, Cuba.
10 USFQ Universidad San Francisco de Quito, Quito, Ecuador.
11 ITESM Tecnológico de Monterrey, Nuevo León, Mexico.
12 UAM Universidad Autónoma Metropolitana-Xochimilco, Ciudad de México, México.
13 IPN Instituto Politécnico Nacional, CIIDIR Unidad Durango, Academia de Genómica, Durango, México.
14 Former: UNAN Universidad Autónoma de Nicaragua, León, Nicaragua; Present: Facultad de Odontología, Universidad Americana, Managua, Nicaragua.
15 UNAN Universidad Autónoma de Nicaragua, León, Nicaragua.
16 UC Universidade de Coimbra, Coimbra, Portugal.
17 UEX, University of Extremadura, Faculty of Medicine and Heath Sciences, Badajoz, Spain.
18 INUBE Instituto Universitario de Investigación Biosanitaria de Extremadura, Badajoz, Spain. Unit Pharmacogenetics HUB SES, Servicio Extremeño de Salud, Badajoz, Spain.
19 Former: Universidad de Montevideo, Montevideo, Uruguay; Present: Goes 2036/Montevideo, Uruguay.

## References

[1] Fricke-Galindo I., Jung-Cook H., Llerena A., López-López M. (2016). Interethnic Variability of Pharmacogenetic Biomarkers in Mexican Healthy Volunteers: A Report from the RIBEF (Ibero-American Network of Pharmacogenetics and Pharmacogenomics). Drug Metab. Pers. Ther..

[2] Naranjo M.-E.G., Rodrigues-Soares F., Peñas-Lledó E.M., Tarazona-Santos E., Fariñas H., Rodeiro I., Terán E., Grazina M., Moya G.E., López-López M. (2018). Interethnic Variability in *CYP2D6*, *CYP2C9*, and *CYP2C19* Genes and Predicted Drug Metabolism Phenotypes Among 6060 Ibero- and Native Americans: RIBEF-CEIBA Consortium Report on Population Pharmacogenomics. OMICS J. Integr. Biol..

[3] Rodrigues-Soares F., Peñas-Lledó E.M., Tarazona-Santos E., Sosa-Macías M., Terán E., López-López M., Rodeiro I., Moya G.E., Calzadilla L.R., Ramírez-Roa R. (2020). Genomic Ancestry, CYP2D6, CYP2C9, and CYP2C19 Among Latin Americans. Clin. Pharmacol. Ther..

[4] Bryc K., Durand E.Y., Macpherson J.M., Reich D., Mountain J.L. (2015). The Genetic Ancestry of African Americans, Latinos, and European Americans across the United States. Am. J. Hum. Genet..

[5] Moreno-Estrada A., Gravel S., Zakharia F., McCauley J.L., Byrnes J.K., Gignoux C.R., Ortiz-Tello P.A., Martínez R.J., Hedges D.J., Morris R.W. (2013). Reconstructing the Population Genetic History of the Caribbean. PLoS Genet..

[6] Castillo L., Diógenes I. (2021). Breve Encuesta Nacional de Autopercepción Racial y Étnica En República Dominicana.

[7] Sosa-Macias M., Moya G.E., Llerena A., Ramírez R., Terán E., Penãs-Lledó E.M., Tarazona-Santos E., Galaviz-Hernández C., Céspedes-Garro C., Acosta H. (2015). Population Pharmacogenetics of Ibero-Latinoamerican Populations (MESTIFAR 2014). Pharmacogenomics.

[8] Céspedes-Garro C., Naranjo M.-E.G., Rodrigues-Soares F., Llerena A., Duconge J., Montané-Jaime L.K., Roblejo H., Fariñas H., Campos M.d.l.A., Ramírez R. (2016). Pharmacogenetic Research Activity in Central America and the Caribbean: A Systematic Review. Pharmacogenomics.

[9] Naranjo M.E.G., de Andrés F., Delgado A., Cobaleda J., Peñas-Lledó E.M., LLerena A. (2016). High Frequency of CYP2D6 Ultrarapid Metabolizers in Spain: Controversy about Their Misclassification in Worldwide Population Studies. Pharmacogenom. J..

[10] de Andrés F., Altamirano-Tinoco C., Ramírez-Roa R., Montes-Mondragón C.F., Dorado P., Peñas-Lledó E.M., LLerena A. (2021). Relationships between CYP1A2, CYP2C9, CYP2C19, CYP2D6 and CYP3A4 Metabolic Phenotypes and Genotypes in a Nicaraguan Mestizo Population. Pharmacogenom. J..

[11] Latinobarometro. https://www.latinobarometro.org/lat.jsp.

[12] Estrada-Veras J.I., Cabrera-Peña G.A., Pérez-Estrella De Ferrán C. (2016). Medical Genetics and Genomic Medicine in the Dominican Republic: Challenges and Opportunities. Mol. Genet. Genom. Med..

[13] Schroeder H., Sikora M., Gopalakrishnan S., Cassidy L.M., Delser P.M., Velasco M.S., Schraiber J.G., Rasmussen S., Homburger J.R., Ávila-Arcos M.C. (2018). Origins and Genetic Legacies of the Caribbean Taino. Proc. Natl. Acad. Sci. USA.

[14] Montinaro F., Busby G.B.J., Pascali V.L., Myers S., Hellenthal G., Capelli C. (2015). Unravelling the Hidden Ancestry of American Admixed Populations. Nat. Commun..

[15] Thornton B.J., Ubiera D.I. (2019). Caribbean Exceptions: The Problem of Race and Nation in Dominican Studies. Lat. Am. Res. Rev..

[16] Dorado P., Cáceres M.C., Pozo-Guisado E., Wong M.L., Licinio J., LLerena A. (2005). Development of a PCR-Based Strategy for CYP2D6 Genotyping Including Gene Multiplication of Worldwide Potential Use. Biotechniques.

[17] Duarte J.D., Thomas C.D., Lee C.R., Huddart R., Agundez J.A.G., Baye J.F., Gaedigk A., Klein T.E., Lanfear D.E., Monte A.A. (2024). Clinical Pharmacogenetics Implementation Consortium Guideline (CPIC) for CYP2D6, ADRB1, ADRB2, ADRA2C, GRK4, and GRK5 Genotypes and Beta-Blocker Therapy. Clin. Pharmacol. Ther..

[18] Cooper-DeHoff R.M., Niemi M., Ramsey L.B., Luzum J.A., Tarkiainen E.K., Straka R.J., Gong L., Tuteja S., Wilke R.A., Wadelius M. (2022). The Clinical Pharmacogenetics Implementation Consortium Guideline for SLCO1B1, ABCG2, and CYP2C9 Genotypes and Statin-Associated Musculoskeletal Symptoms. Clin. Pharmacol. Ther..

[19] Lee C.R., Luzum J.A., Sangkuhl K., Gammal R.S., Sabatine M.S., Stein C.M., Kisor D.F., Limdi N.A., Lee Y.M., Scott S.A. (2022). Clinical Pharmacogenetics Implementation Consortium Guideline for CYP2C19 Genotype and Clopidogrel Therapy: 2022 Update. Clin. Pharmacol. Ther..

[20] Yaeger R., Avila-bront A., Abdul K., Nolan P.C., Grann V.R., Birchette M.G., Choudhry S., Burchard E.G., Beckman K.B., Gorroochurn P. (2008). Comparing Genetic Ancestry and Self-Described Race in African Americans Born in the United States and in Africa. Cancer Epidemiol. Biomark. Prev..

[21] Alexander D.H., Novembre J., Lange K. (2009). Fast Model-Based Estimation of Ancestry in Unrelated Individuals. Genome Res..

[22] Auton A., Abecasis G.R., Altshuler D.M., Durbin R.M., Bentley D.R., Chakravarti A., Clark A.G., Donnelly P., Eichler E.E., Flicek P. (2015). A Global Reference for Human Genetic Variation. Nature.

[23] Jombart T. (2008). Adegenet: A R Package for the Multivariate Analysis of Genetic Markers. Bioinformatics.

[24] R: The R Project for Statistical Computing. https://www.r-project.org/.

[25] La AEMPS Lanza Una Base de Datos de Biomarcadores Farmacogenómicos. https://www.aemps.gob.es/informa/la-aemps-lanza-una-base-de-datos-de-biomarcadores-farmacogenomicos-en-fichas-tecnicas-de-medicamentos/#.

[26] Table of Pharmacogenetic Associations|FDA. https://www.fda.gov/medical-devices/precision-medicine/table-pharmacogenetic-associations.

[27] Multidisciplinary: Pharmacogenomics|European Medicines Agency (EMA). https://www.ema.europa.eu/en/human-regulatory-overview/research-development/scientific-guidelines/multidisciplinary-guidelines/multidisciplinary-pharmacogenomics.

[28] CPIC. https://cpicpgx.org/.

[29] Johnson J., Caudle K., Gong L., Whirl-Carrillo M., Stein C., Scott S., Lee M., Gage B., Kimmel S., Perera M. (2017). Clinical Pharmacogenetics Implementation Consortium (CPIC) Guideline for Pharmacogenetics-Guided Warfarin Dosing: 2017 Update. Clin. Pharmacol. Ther..

[30] PharmGKB. https://www.pharmgkb.org/.

[31] Llerena A., Alvarez M., Dorado P., González I., Peñas-LLedó E., Pérez B., Cobaleda J., Calzadilla L.R. (2014). Interethnic Differences in the Relevance of CYP2C9 Genotype and Environmental Factors for Diclofenac Metabolism in Hispanics from Cuba and Spain. Pharmacogenom. J..

[32] Hicks J.K., Sangkuhl K., Swen J.J., Ellingrod V.L., Müller D.J., Shimoda K., Bishop J.R., Kharasch E.D., Skaar T.C., Gaedigk A. (2017). Clinical Pharmacogenetics Implementation Consortium Guideline (CPIC) for CYP2D6 and CYP2C19 Genotypes and Dosing of Tricyclic Antidepressants: 2016 Update. Clin. Pharmacol. Ther..

[33] Bousman C.A., Stevenson J.M., Ramsey L.B., Sangkuhl K., Hicks J.K., Strawn J.R., Singh A.B., Ruaño G., Mueller D.J., Tsermpini E.E. (2023). Clinical Pharmacogenetics Implementation Consortium (CPIC) Guideline for CYP2D6, CYP2C19, CYP2B6, SLC6A4, and HTR2A Genotypes and Serotonin Reuptake Inhibitor Antidepressants. Clin. Pharmacol. Ther..

[34] Magalhães P., Alves G., Llerena A., Falcão A. (2014). Venlafaxine Pharmacokinetics Focused on Drug Metabolism and Potential Biomarkers. Drug Metab. Drug Interact..

[35] Berecz R., LLerena A., De la Rubia A., Gómez J., Kellermann M., Dorado P., Degrell I. (2002). Relationship between Risperidone and 9-Hydroxy-Risperidone Plasma Concentrations and CYP2D6 Enzyme Activity in Psychiatric Patients. Pharmacopsychiatry.

[36] Peñas-Lledó E.M., Guillaume S., de Andrés F., Cortés-Martínez A., Dubois J., Kahn J.P., Leboyer M., Olié E., LLerena A., Courtet P. (2022). A One-Year Follow-up Study of Treatment-Compliant Suicide Attempt Survivors: Relationship of CYP2D6-CYP2C19 and Polypharmacy with Suicide Reattempts. Transl. Psychiatry.

[37] Jukić M.M., Opel N., Ström J., Carrillo-Roa T., Miksys S., Novalen M., Renblom A., Sim S.C., Peñas-Lledó E.M., Courtet P. (2017). Elevated CYP2C19 Expression Is Associated with Depressive Symptoms and Hippocampal Homeostasis Impairment. Mol. Psychiatry.

[38] Rodríguez-Antona C., Gurwitz D., de Leon J., Llerena A., Kirchheiner J., de Mesa E.G., Ibarreta D. (2009). CYP2D6 Genotyping for Psychiatric Patients Treated with Risperidone: Considerations for Cost-Effectiveness Studies. Pharmacogenomics.

[39] Peñas-LLedó E., LLerena A. (2023). Clinical Use of Pre-Emptive Pharmacogenetic Programmes. Lancet.

[40] De Andrés F., Terán S., Hernández F., Terán E., Llerena A. (2016). To Genotype or Phenotype for Personalized Medicine? CYP450 Drug Metabolizing Enzyme Genotype-Phenotype Concordance and Discordance in the Ecuadorian Population. OMICS J. Integr. Biol..

[41] de Andrés F., Sosa-Macías M., Ramos B.P.L., Naranjo M.E.G., LLerena A. (2017). CYP450 Genotype/Phenotype Concordance in Mexican Amerindian Indigenous Populations-Where to from Here for Global Precision Medicine?. OMICS J. Integr. Biol..

[42] Shah R.R., Gaedigk A., Llerena A., Eichelbaum M., Stingl J., Smith R.L. (2016). CYP450 Genotype and Pharmacogenetic Association Studies: A Critical Appraisal. Pharmacogenomics.

[43] Sosa-Macías M., Fricke-Galindo I., Fariñas H., Monterde L., Ruiz-Cruz E.D., Molina-Guarneros J., Tarazona-Santos E., Rodrigues-Soares F., Galaviz-Hernández C., Peñas-Lledó E. (2023). Pharmacogenetics: Ethnicity, Treatment and Health in Latin American Populations. Pharmacogenomics.

[44] Sosa-Macías M., Teran E., Waters W., Fors M.M., Altamirano C., Jung-Cook H., Galaviz-Hernández C., López-López M., Remírez D., Moya G.E. (2016). Pharmacogenetics and Ethnicity: Relevance for Clinical Implementation, Clinical Trials, Pharmacovigilance and Drug Regulation in Latin America. Pharmacogenomics.

[45] Peñas-LLedó E., Terán E., Sosa-Macías M., Galaviz-Hernández C., Gil J.P., Nair S., Diwakar S., Hernández I., Lara-Riegos J., Ramírez-Roa R. (2020). Challenges and Opportunities for Clinical Pharmacogenetic Research Studies in Resource-Limited Settings: Conclusions From the Council for International Organizations of Medical Sciences-Ibero-American Network of Pharmacogenetics and Pharmacogenomics Meeting. Clin. Ther..

